# Serum *N* ^*1*^‐Methylnicotinamide is Associated With Coronary Artery Disease in Chinese Patients

**DOI:** 10.1161/JAHA.116.004328

**Published:** 2017-02-08

**Authors:** Ming Liu, Jihong Chu, Yang Gu, Haibo Shi, Rusheng Zhang, Lingzhun Wang, Jiandong Chen, Le Shen, Peng Yu, Xiaohu Chen, Wenzheng Ju, Zhenxing Wang

**Affiliations:** ^1^ Department of Cardiology Jiangsu Province Hospital of Traditional Chinese Medicine The Affiliated Hospital of Nanjing University of Chinese Medicine Nanjing Jiangsu China; ^2^ Department of Clinical Pharmacology Jiangsu Province Hospital of Traditional Chinese Medicine The Affiliated Hospital of Nanjing University of Chinese Medicine Nanjing Jiangsu China; ^3^ Institute of Hypertension Jiangsu Province Hospital of Traditional Chinese Medicine The Affiliated Hospital of Nanjing University of Chinese Medicine Nanjing Jiangsu China

**Keywords:** coronary angiography, coronary artery disease, inflammation, *N*^*1*^‐methylnicotinamide, Coronary Artery Disease, Inflammation

## Abstract

**Background:**

We previously reported that serum *N* ^*1*^‐methylnicotinamide (me‐Nam), an indicator of nicotinamide N‐methyltransferase activity, is associated with obesity and diabetes mellitus in Chinese patients. However, whether nicotinamide N‐methyltransferase plays a role in human coronary artery disease (CAD) remains to be elucidated. We aim to investigate the associations of serum me‐Nam with CAD in Chinese patients.

**Methods and Results:**

Serum me‐NAM was measured by liquid chromatography‐mass spectrometry in patients with (n=230) or without (n=103) CAD as defined by coronary angiography. The severity of CAD was expressed by number of diseased coronary arteries. Serum me‐Nam was higher (7.65 ng/mL versus 4.95 ng/mL,* P*<0.001) in patients with CAD than in those without. Serum me‐Nam was positively correlated with high‐sensitivity C‐reactive protein and negatively correlated with high‐density lipoprotein before and after adjustment for potential confounding variables (*P*≤0.002). In multivariable logistic regression analyses, compared with those in the lowest tertile of serum me‐NAM levels, patients in the top tertile had the highest risks for CAD (odds ratio, 4.21; 95% CI, 1.97–8.97 [*P*<0.001]). After adjustment for potential confounding variables, serum me‐NAM was also increased from 0‐ to 3‐vessel disease (*P* for trend=0.01).

**Conclusions:**

Serum me‐Nam is strongly associated with presence and severity of CAD, suggesting nicotinamide N‐methyltransferase as a potential target for treating atherosclerosis in humans.

## Introduction

Nicotinamide N‐methyltransferase (NNMT) gene is abundantly expressed in adipose tissue and liver and lower expressed in other organs.^1^, ^2^ NNMT behaves as an important methyltransferase to catalyze the transfer of a methyl group from s‐adenosylmethionine to nicotinamide (vitamin B3) to generate *N* ^*1*^‐methylnicotinamide (me‐NAM) and s‐adenosylhomocysteine, a precursor of homocysteine,^1^, ^2^ and is therefore considered to play a unique role in the development of coronary artery disease (CAD) because it has long been known that elevated homocysteine is associated with cardiovascular disease.^3^

Recent studies have suggested that NNMT activity was increased in obesity and type 2 diabetes mellitus in mice and humans.^4^, ^5^, ^6^ Our recent epidemiological study also revealed that serum me‐NAM, an indicator of NNMT activity, was associated with obesity and diabetes mellitus in a Chinese population.^7^ Because obesity and diabetes mellitus were independent risk factors for the development of CAD,^8^, ^9^ we hypothesized that increased serum me‐NAM levels might be associated with CAD. Indeed, upregulation of hepatic NNMT activity and subsequent increase in plasma me‐NAM levels were associated with the progression of atherosclerosis in an animal model.^10^ Evidence for the association of me‐NAM with CAD is still lacking in humans. Therefore, the present case‐control study aims to investigate the relationship between serum me‐NAM levels and CAD in Chinese patients.

## Methods

### Study Population

From April 2015 to July 2015, we invited 460 consecutive patients who were suspected of having CAD with chest pain and were referred for diagnostic coronary angiography to the Department of Cardiology, Jiangsu Province Hospital of Traditional Chinese Medicine, Nanjing, China. Of the 460 invited, 342 (74.3%) participated. We excluded 9 patients from the present analyses because of inadequate blood samples for me‐NAM measurements (n=2) or missing information on angiographic data (n=2), plasma glucose (n=2), or serum lipids (n=3). Thus, the total number of participants in the present analyses was 333. All patients gave written informed consent. The study protocol was approved by the ethics committee of Jiangsu Province Hospital of Traditional Chinese Medicine.

### Anthropometric and Biochemical Measurements

Sociodemographics, medical history, smoking and drinking habits, and the use of medications were documented with a standardized questionnaire. Body weight, body height, and blood pressure (BP) were measured by an experienced nurse. Body mass index (BMI) was calculated as weight in kilograms divided by height in meters squared. Three sitting BP measurements taken consecutively at 5‐minutes intervals using an automated electronic device (Omron HEM 7130, Kyoto, Japan) were averaged for analysis. Venous blood samples were taken after overnight fasting for the measurement of plasma glucose, serum creatinine, total cholesterol, high‐density lipoprotein (HDL) cholesterol, low‐density lipoprotein (LDL) cholesterol, triglycerides (TGs), alanine aminotransferase (ALT), γ‐glutamyltransferase (GGT), and high‐sensitivity C‐reactive protein (hs‐CRP). Serum samples were subsequently stored in aliquots at −80°C for measurements of me‐NAM.

As previously described,^7^ serum me‐NAM concentrations were determined by liquid chromatography with tandem mass spectrometry (LC/MS/MS, Agilent 6430 Triple Quad LC/MS, USA) in the Clinical Pharmacology Laboratory, Jiangsu Province Hospital of Traditional Chinese Medicine. The intra‐ and inter‐assay coefficients of variance were 3.69% and 4.95% for me‐NAM, respectively. The analytic sensitivity of the assays for measuring me‐NAM was 2.5 ng/mL. See Data S1 for further details (Tables S1 through S3).

Hypertension was defined as a BP of at least 140 mm Hg systolic or 90 mm Hg diastolic or as the use of antihypertensive drugs. Diabetes mellitus was defined as a fasting plasma glucose of at least 7.0 mmol/L or as the use of antidiabetic agents. Dyslipidemia was defined according to guideline of the National Cholesterol Education Program (Adult Treatment Panel III).^11^

### Coronary Angiography and Analysis

Coronary angiography was performed via the radial or femoral access with 6‐F diagnostic catheters. The angiographic assessment was performed independently by two interventional cardiologists (H.B.S., R.S.Z.) who were both blinded to the concentrations of me‐NAM, according to lesion classification scheme of the American College of Cardiology/American Heart Association.^12^ In case of disagreement, the difference in interpretation was resolved by a third reviewer (Z.X.W.). CAD was defined as luminal diameter narrowing estimated visually at least 50% in any epicardial coronary artery. We then defined 3 patient groups based on the degrees of CAD: single‐vessel disease, double‐vessel disease, and triple‐vessel disease.

### Statistical Methods

Statistical analyses were performed using SAS software, version 9.1 (SAS institute, Cary, NC). Data were presented as mean±SD or median (25th and 75th percentiles) for continuous variables or as percentage for categorical variables. Departure from normality was tested by the Shapiro‐Wilk statistic. Serum me‐NAM, TGs, ALT, GGT, and hs‐CRP concentrations were not normally distributed and were, therefore, logarithmically transformed for statistical analyses. Means and proportions were compared with the Student *t* test and Fisher exact test, respectively. Relationships among me‐NAM, age, BMI, BP, liver enzymes, renal function, fasting plasma glucose, serum lipids, and hs‐CRP were examined by calculation of partial correlation coefficients. Multivariable logistic regression analyses were employed to evaluate the odds ratios (ORs) and 95% CIs of having CAD for higher tertiles of me‐NAM compared with the lowest tertile. Linear regression analysis was used to test for trend of the changes of serum me‐NAM concentrations across the severity of coronary angiography (normal to triple‐vessel disease). Comparisons between groups were performed with ANOVA, and Bonferroni correction was used for post hoc analysis. Sensitivity analyses stratified by sex or after exclusion of diabetic patients were performed. A 2‐sided value of *P*<0.05 was considered statistically significant.

## Results

### Characteristics of the Study Participants

The 333 participants (mean age 65.6±10.6 years) included 193 men (57.9%), and 233 had hypertension (70.0%), 103 had type 2 diabetes mellitus (30.9%), and 173 had dyslipidemia (52.0%). Table 1 summarizes the characteristics of the study participants with or without CAD. There were no statistically significant differences between CAD and control patients with respect to BMI, systolic/diastolic BP, rates of alcohol intake, plasma glucose, total and LDL cholesterol, and TG, ALT and GGT, and hs‐CRP levels. However, CAD patients, compared with control patients, had higher proportions of male sex and current smoking (*P*≤0.02); higher rates of hypertension or taking antihypertensive drugs, diabetes mellitus or taking antihyperglycemic drugs, and dyslipidemia or taking hypolipidemic drugs (*P*≤0.001); and higher serum creatinine and lower serum HDL cholesterol concentrations (*P*≤0.03). In addition, CAD patients, compared with controls, had higher serum me‐NAM concentrations (7.65 ng/mL versus 4.95 ng/mL; *P*<0.001).

**Table 1:** Characteristics of Control Subjects and Patients With CAD

Characteristic	Control (n=103)	CAD Patients (n=230)	*P* Value
Age, y	61.8±9.7	67.2±10.5	<0.001
Male, n (%)	50 (48.5)	143 (62.2)	0.02
Body mass index, kg/m^2^	24.7±3.5	24.9±3.3	0.65
Systolic blood pressure, mm Hg	134.3±18.8	137.9±17.9	0.10
Diastolic blood pressure, mm Hg	81.7±11.5	81.4±11.7	0.84
Current smoking, n (%)	18 (17.5)	73 (31.7)	0.007
Alcohol intake, n (%)	17 (16.5)	57 (24.8)	0.09
Hypertension, n (%)	56 (54.4)	177 (77.0)	<0.001
Diabetes mellitus, n (%)	12 (11.7)	91 (39.6)	<0.001
Dyslipidemia, n (%)	37 (35.9)	136 (59.1)	<0.001
Taking antihypertensive drugs, n (%)	47 (45.6)	159 (69.1)	<0.001
Taking antihyperglycemic drugs, n (%)	11 (10.7)	70 (30.4)	<0.001
Taking hypolipidemic drugs, n (%)	20 (19.4)	86 (37.4)	0.001
Plasma glucose, mmol/L	5.50±1.40	5.85±1.70	0.07
Total cholesterol, mmol/L	4.10±0.77	4.13±1.16	0.80
LDL cholesterol, mmol/L	2.21±0.58	2.29±0.75	0.36
HDL cholesterol, mmol/L	1.24±0.31	1.16±0.33	0.03
Triglycerides, mmol/L	1.04 (0.80–1.75)	1.23 (0.88–1.69)	0.40
Alanine aminotransferase, U/L	19 (15–27)	20 (15–33)	0.13
γ‐glutamyltransferase, U/L	19 (13–35)	21 (15–33)	0.91
Serum creatinine, μmol/L	76.9±16.9	86.9±20.3	<0.001
High‐sensitivity C‐reactive protein, mg/L^a^	5.0 (3.0–11.0)	6.0 (2.5–12.0)	0.88
*N* ^*1*^‐methylnicotinamide, ng/mL	4.95 (3.31–8.74)	7.65 (4.57–11.59)	<0.001

Data are mean ± standard deviation, median with interquartile range in parenthesis, or number with percentage in parenthesis. CAD indicates coronary artery disease; LDL, low‐density lipoprotein; HDL, high‐density lipoprotein.

a High‐sensitivity C‐reactive protein was measured in 71 control and 60 CAD patients.

### Association of me‐NAM With Clinical Parameters

We next investigated the relationship of serum me‐NAM levels with various anthropometric and laboratory parameters. Serum me‐NAM concentrations were positively associated with BMI (*r*=0.14; *P*=0.03) and hs‐CRP (*r*=0.39; *P*<0.001) but negatively associated with HDL cholesterol (*r*=−0.17; *P*=0.002) in the CAD group. All of these correlations remained statistically significant (*P*≤0.01) after adjustments for age, current smoking, alcohol intake, BMI, systolic BP, serum ALT and GGT, creatinine, fasting plasma glucose, total and LDL cholesterol, and TGs, as appropriate. In the control group, there was no correlation between me‐NAM and these clinical parameters.

### Association of me‐NAM With Presence and Severity of CAD

In logistic regression analyses, compared with patients in the lowest tertile of serum me‐NAM levels, patients in the highest tertile were associated with an increased OR of CAD both before and after adjustment for potential confounders (ie, age; sex; BMI; systolic BP; current smoking and alcohol intake; hypertension; diabetes mellitus; dyslipidemia; use of antihypertensive, antihyperglycemic, and hypolipidemic drugs; and fasting plasma glucose, total and HDL cholesterol, and TGs) (Table 2). Similar results were obtained by logistic regression using log‐transformed me‐NAM as a continuous variable (data not show).

**Table 2:** Associations of Serum me‐NAM Concentration Tertiles With CAD

	Serum me‐NAM, ng/mL (Tertile 2 vs Tertile 1)		Serum me‐NAM, ng/mL (Tertile 3 vs Tertile 1)	
	Odds Ratio (95% CI)	*P* Value	Odds Ratio (95% CI)	*P* Value
Crude model	2.06 (1.18–3.59)	0.01	3.61 (1.97–6.61)	<0.001
Adjusted model	2.41 (1.23–4.72)	0.01	4.21 (1.97–8.97)	<0.001

In the adjusted model, odds ratio (95% CI) were adjusted for age, sex, body mass index, systolic blood pressure, current smoking and alcohol intake, hypertension, diabetes mellitus, dyslipidemia, use of antihypertensive, antihyperglycemic and hypolipidemic drugs, and fasting plasma glucose, total and low‐density lipoprotein cholesterol, and triglycerides. CAD indicates coronary artery disease; me‐NAM, *N* ^1^‐Methylnicotinamide.

We next performed linear regression analysis to investigate whether me‐NAM levels were associated with severity of CAD. In unadjusted analyses, serum me‐NAM concentrations were increased across the severity of CAD (Table 3). After adjustment for the aforementioned covariates, the trends of serum me‐NAM concentrations across the severity of CAD remained statistically significant (Figure and Table 3).

**Table 3:** Associations of Serum me‐NAM With the Severity of CAD

	Severity of CAD				*P* for ANOVA
	Normal	1‐Vessel	2‐Vessel	3‐Vessel	
Serum me‐NAM, ng/mL					
Crude model	6.04±0.89	8.70±0.87*	10.02±1.01†	10.55±1.02†	0.01
Adjusted model	6.15±0.80	8.56±0.84*	9.53±1.05†	10.95±0.92‡	0.001

Values are mean±standard error. In adjusted analysis, age, sex, body mass index, systolic blood pressure, current smoking and alcohol intake, hypertension, diabetes mellitus, dyslipidemia, use of antihypertensive, antihyperglycemic and hypolipidemic drugs, and fasting plasma glucose, total and HDL cholesterol, and triglycerides were considered as covariables. CAD indicates coronary artery disease; Me‐NAM, *N* ^*1*^‐methylnicotinamide.

*P<0.05; ^†^ *P*<0.01; ^‡^ *P*<0.001, vs normal.

**Figure 1:**
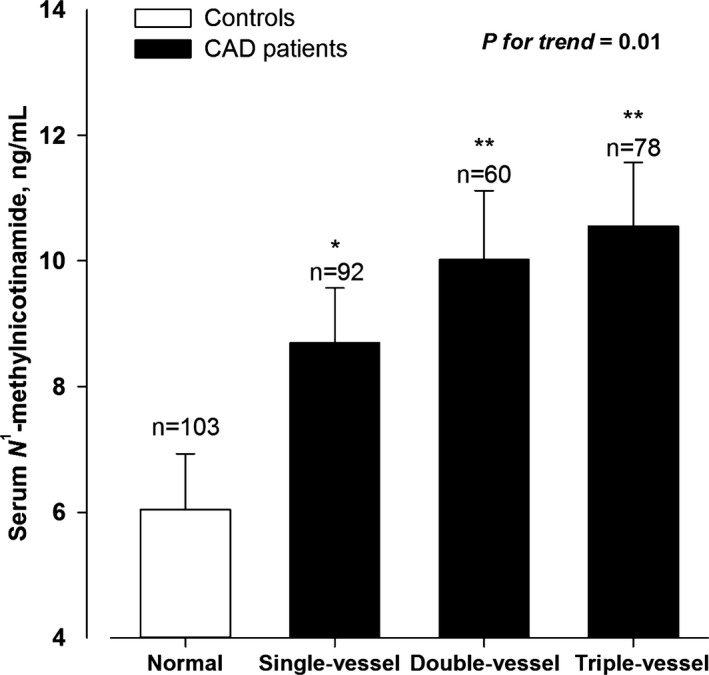
Association between serum *N* ^1^‐methylnicotinamide (me‐NAM) and severity of coronary artery disease (CAD). The analysis was adjusted for age, sex, body mass index, systolic blood pressure, current smoking and alcohol intake, hypertension, diabetes mellitus, dyslipidemia, use of antihypertensive, antihyperglycemic and hypolipidemic drugs, and fasting plasma glucose, total and high‐density lipoprotein (HDL) cholesterol, and triglycerides. The *P* value for test for trend of the changes of serum me‐NAM concentrations across the severity of coronary angiography is given. *P<0.01 vs normal. **P<0.001 vs normal.

Separating all patients into men and women did not materially change the results (Table S4 and Figure S1). In further analyses, similar results were observed when we performed analyses in 230 patients without diabetes. Indeed, after exclusion of diabetic patients, patients in the highest tertile compared with the lowest tertile of serum me‐NAM were associated with higher risks for CAD after adjustment for potential confounders (OR, 4.45; 95% CI, 1.95–10.15 [*P*<0.001]). Similarly, the trends of serum me‐NAM concentrations across the severity of CAD remained statistically significant after adjustment for potential confounders (*P*<0.001).

## Discussion

The main finding of our study is that serum me‐NAM, an indicator of NNMT activity, is strongly associated with CAD, independent of conventional CAD risk factors. Among 333 Chinese adults in our study, patients in the highest compared with those in the lowest tertile of serum me‐NAM were associated with a ≈3‐fold increase in the risk for CAD. Another interesting finding of the present study is that me‐NAM concentrations are also associated with severity of CAD.

Until now, there has been only one animal‐based study that examined the effects of hepatic NNMT activity and plasma me‐NAM levels in the development of atherosclerosis.^10^ Mateuszuk et al^10^ found that hepatic NNMT activity and plasma me‐NAM levels increased by more than 2‐fold in apolipoprotein‐E/LDL double knockout mice compared with controls, and they were associated with the progression of atherosclerosis. These findings support our results that serum me‐NAM concentrations were not only associated with the risk for CAD, but also with the severity of CAD. However, it is not clear whether increased me‐NAM concentrations is a compensatory response or only a marker of atherosclerosis. Notably, although traditionally considered as an inactive or toxic metabolite of terminal nicotinamide clearance,^13^, ^14^ some publications suggest that, at pharmacological doses, me‐NAM has biological activity and affects endothelial cell function.^15^, ^16^, ^17^ Recently, Domagala et al^18^ reported that me‐NAM ameliorated endothelial dysfunction via increasing endothelial NO synthase–mediated NO release from endothelial cells. In addition, Mateuszuk et al^19^ demonstrated that me‐NAM displayed antiatherosclerotic effects in apolipoprotein‐E/LDL double knockout mice via improving endothelial function, inhibiting platelet activation and inflammation. Therefore, we speculate that the increase in serum me‐NAM in CAD might be attributable to a defensive response, which may represent an ability to adapt to atherosclerosis by preventing endothelial dysfunction. However, we cannot exclude that the elevated me‐NAM levels may be associated with other unknown factors of CAD, such as diabetes mellitus,^7^ which was widely recognized as a CAD risk equivalent.^20^ Indeed, Kannt's and our recent studies reported a significant association between me‐NAM and diabetes mellitus.^5^, ^7^ To exclude the role of diabetes mellitus on the relationship between me‐NAM and atherosclerosis, we further investigated the association of me‐NAM with CAD in nondiabetic patients. Although there was no statistical difference, the effect of me‐NAM on CAD was slightly larger after exclusion of diabetes mellitus (OR, 4.45 versus 4.21). Further studies are needed to delineate the mechanisms and independent role of me‐NAM in CAD and atherosclerosis. Of note, serum me‐NAM levels in the current study was slightly lower than that in our previous study. Although the exact reasons for the differences are unclear, several potential factors including study design, sample size, ethnicity and patient characteristics may explain the differences.

Our finding on the association between serum me‐NAM concentrations and HDL is consistent with the result of our previous epidemiological study^7^ but not with a recent study.^21^ Hong et al^21^ reported that serum me‐NAM was not significantly associated with HDL. The disparities may be due to the different sample size in Hong's study (n=51) compared with ours (n=333), and the patients in Hong's study were of different ethnicity and those with morbid obesity who needed elective gastric bypass surgery. Hong's study did not find the association between me‐NAM and HDL perhaps due to the severity of obese patients because serum me‐NAM levels were affected by obesity.^5^, ^7^ However, hepatic NNMT activity was inversely associated with HDL in an animal model in their study,^21^ which is consistent with our finding.

Another interesting observation of the current study is a positive correlation between me‐NAM and hs‐CRP, independent of other risk factors, which indicates that the association between me‐NAM and CAD is probably through inflammatory pathways because hs‐CRP, a well‐known marker of inflammation,^22^ may contribute to the development of CAD.^23^ Indeed, hepatic NNMT activity and me‐NAM plasma concentrations increased with the development of vascular inflammation in the aorta.^10^ However, we cannot rule out that increased me‐NAM concentrations in our study may represent a compensatory mechanism in response to inflammation because me‐NAM possesses anti‐inflammatory properties by inhibiting the generation of reactive oxygen species.^24^

## Study Limitations

Our study should be interpreted within the context of its limitations. The cross‐sectional design does not allow causal inference. In addition, we only investigated me‐NAM levels in Chinese patients, and therefore our findings need to be confirmed in other ethnicities.

## Conclusions

Our first case‐control study shows significant associations of serum me‐NAM with the presence and severity of CAD in Chinese patients. Although prospective and interventional studies are necessary to investigate whether elevated NNMT activity may play a causal role in CAD in humans, our findings provide novel insights into the potential role of NNMT in CAD.

## Sources of Funding

The present study was in part supported by grants from National Natural Science Foundation of China (grant numbers 81273943 and 81573908) to Xiao‐hu Chen, and the National Natural Science Foundation of China (grant number 81573685) to Ju.

## Disclosures

None.

## Supporting information


**Data S1.** Supplemental methods.
**Table S1.** HPLC Conditions for Measuring *N* ^*1*^‐Methylnicotinamide
**Table S2.** MS Conditions for Measuring *N* ^*1*^‐Methylnicotinamide
**Table S3.** Stability of *N* ^*1*^‐Methylnicotinamide (me‐NAM) Under Different Storage Conditions (n=3)
**Table S4.** Associations of Serum *N* ^*1*^‐Methylnicotinamide Concentration Tertiles With Coronary Artery Disease by Sex
**Figure S1.** Association between serum *N* ^*1*^‐methylnicotinamide and severity of coronary artery disease in men (left) and women (right), respectively.

## References

[1] Aksoy S , Szumlanski CL , Weinshilboum RM . Human liver nicotinamide N‐methyltransferase. cDNA cloning, expression, and biochemical characterization. J Biol Chem. 1994;269:14835–14840. 8182091

[2] Riederer M , Erwa W , Zimmermann R , Frank S , Zechner R . Adipose tissue as a source of nicotinamide N‐methyltransferase and homocysteine. Atherosclerosis. 2009;204:412–417. 18996527 10.1016/j.atherosclerosis.2008.09.015

[3] Shai I , Stampfer MJ , Ma J , Manson JE , Hankinson SE , Cannuscio C , Selhub J , Curhan G , Rimm EB . Homocysteine as a risk factor for coronary heart diseases and its association with inflammatory biomarkers, lipids and dietary factors. Atherosclerosis. 2004;177:375–381. 15530913 10.1016/j.atherosclerosis.2004.07.020

[4] Kraus D , Yang Q , Kong D , Banks AS , Zhang L , Rodgers JT , Pirinen E , Pulinilkunnil TC , Gong F , Wang YC , Cen Y , Sauve AA , Asara JM , Peroni OD , Monia BP , Bhanot S , Alhonen L , Puigserver P , Kahn BB . Nicotinamide N‐methyltransferase knockdown protects against diet‐ induced obesity. Nature. 2014;508:258–262. 24717514 10.1038/nature13198 PMC4107212

[5] Kannt A , Pfenninger A , Teichert L , Tonjes A , Dietrich A , Schon MR , Kloting N , Bluher M . Association of nicotinamide‐N‐methyltransferase mRNA expression in human adipose tissue and the plasma concentration of its product, 1‐methylnicotinamide, with insulin resistance. Diabetologia. 2015;58:799–808. 25596852 10.1007/s00125-014-3490-7 PMC4351435

[6] Salek RM , Maguire ML , Bentley E , Rubtsov DV , Hough T , Cheeseman M , Nunez D , Sweatman BC , Haselden JN , Cox RD , Connor SC , Griffin JL . A metabolomic comparison of urinary changes in type 2 diabetes in mouse, rat, and human. Physiol Genomics. 2007;29:99–108. 17190852 10.1152/physiolgenomics.00194.2006

[7] Liu M , Li LH , Chu JH , Zhu BY , Zhang QT , Yin XY , Jiang WM , Dai GL , Ju WZ , Wang ZX , Yang Q , Fang ZY . Serum N^1^‐methylnicotinamide is associated with obesity and diabetes in Chinese. J Clin Endocrinol Metab. 2015;100:3112–3117. 26066674 10.1210/jc.2015-1732 PMC4525009

[8] Romero‐Corral A , Montori VM , Somers VK , Korinek J , Thomas RJ , Allison TG , Mookadam F , Lopez‐Jimenez F . Association of bodyweight with total mortality and with cardiovascular events in coronary artery disease: a systematic review of cohort studies. Lancet. 2006;368:666–678. 16920472 10.1016/S0140-6736(06)69251-9

[9] Beckman JA , Creager MA , Libby P . Diabetes and atherosclerosis: epidemiology, pathophysiology, and management. JAMA. 2002;287:2570–2581. 12020339 10.1001/jama.287.19.2570

[10] Mateuszuk L , Khomich TI , Slominska E , Gajda M , Wojcik L , Lomnicka M , Gwozdz P , Chlopicki S . Activation of nicotinamide N‐methyltrasferase and increased formation of 1‐methylnicotinamide (MNA) in atherosclerosis. Pharmacol Rep. 2009;61:76–85. 19307695 10.1016/s1734-1140(09)70009-x

[11] Grundy SM , Cleeman JI , Merz CN , Brewer HB Jr , Clark LT , Hunninghake DB , Pasternak RC , Smith SC Jr , Stone NJ ; National Heart, Lung, and Blood Institute; American College of Cardiology Foundation; American Heart Association . Implications of recent clinical trials for the National Cholesterol Education Program Adult Treatment Panel III guidelines. Circulation. 2004;110:227–239. 15249516 10.1161/01.CIR.0000133317.49796.0E

[12] Ellis SG , Vandormael MG , Cowley MJ , DiSciascio G , Deligonul U , Topol EJ , Bulle TM . Coronary morphologic and clinical determinants of procedural outcome with angioplasty for multivessel coronary disease. Implications for patient selection. Multivessel Angioplasty Prognosis Study Group. Circulation. 1990;82:1193–1202. 2401060 10.1161/01.cir.82.4.1193

[13] Maïza A , Waldek S , Ballardie FW , Daley‐Yates PT . Estimation of renal tubular secretion in man, in health and disease, using endogenous N‐1‐methylnicotinamide. Nephron. 1992;60:12–16. 1531378 10.1159/000186698

[14] Delaney J , Hodson MP , Thakkar H , Connor SC , Sweatman BC , Kenny SP , McGill PJ , Holder JC , Hutton KA , Haselden JN , Waterfield CJ . Tryptophan‐NAD+ pathway metabolites as putative biomarkers and predictors of peroxisome proliferation. Arch Toxicol. 2005;79:208–223. 15838709 10.1007/s00204-004-0625-5

[15] Chopicki S , Swies J , Mogielnicki A , Buczko W , Bartus M , Lomnicka M , Adamus J , Gebicki J . 1‐Methylnicotinamide (MNA), a primary metabolite of nicotinamide, exerts anti‐thrombotic activity mediated by a cyclooxygenase‐2/prostacyclin pathway. Br J Pharmacol. 2007;152:230–239. 17641676 10.1038/sj.bjp.0707383 PMC1978255

[16] Bryniarski K , Biedron R , Jakubowski A , Chlopicki S , Marcinkiewicz J . Anti‐inflammatory effect of 1‐methylnicotinamide in contact hypersensitivity to oxazolone in mice; involvement of prostacyclin. Eur J Pharmacol. 2008;578:332–338. 17935712 10.1016/j.ejphar.2007.09.011

[17] Bartuś M , Łomnicka M , Kostogrys RB , Kaźmierczak P , Watała C , Słominska EM , Smoleński RT , Pisulewski PM , Adamus J , Gebicki J , Chlopicki S . 1‐Methylnicotinamide (MNA) prevents endothelial dysfunction in hypertriglyceridemic and diabetic rats. Pharmacol Rep. 2008;60:127–138. 18276994

[18] Domagala TB , Szeffler A , Dobrucki LW , Dropinski J , Polanski S , Leszczynska‐Wiloch M , Kotula‐Horowitz K , Wojciechowski J , Wojnowski L , Szczeklik A , Kalinowski L . Nitric oxide production and endothelium‐dependent vasorelaxation ameliorated by N1‐methylnicotinamide in human blood vessels. Hypertension. 2012;21:825–832. 10.1161/HYPERTENSIONAHA.111.183210 22353616

[19] Mateuszuk L , Jasztal A , Maslak E , Gasior‐Glogowska M , Baranska M , Sitek B , Kostogrys R , Zakrzewska A , Kij A , Walczak M , Chlopicki S . Antiatherosclerotic effects of 1‐methylnicotinamide in apolipoprotein E/low‐density lipoprotein receptor‐deficient mice: a comparison with nicotinic acid. J Pharmacol Exp Ther. 2016;356:514–524. 26631491 10.1124/jpet.115.228643 PMC6047228

[20] Haffner SM , Lehto S , Rönnemaa T , Pyörälä K , Laakso M . Mortality from coronary heart disease in subjects with type 2 diabetes and in nondiabetic subjects with and without prior myocardial infarction. N Engl J Med. 1998;339:229–234. 9673301 10.1056/NEJM199807233390404

[21] Hong S , Moreno‐Navarrete JM , Wei X , Kikukawa Y , Tzameli I , Prasad D , Lee Y , Asara JM , Fernandez‐Real JM , Maratos‐Flier E , Pissios P . Nicotinamide N‐methyltransferase regulates hepatic nutrient metabolism through Sirt1 protein stabilization. Nat Med. 2015;21:887–894. 26168293 10.1038/nm.3882 PMC4529375

[22] Anderson JL , Carlquist JF , Muhlestein JB , Horne BD , Elmer SP . Evaluation of C‐reactive protein, an inflammatory marker, and infectious serology as risk factors for coronary artery disease and myocardial infarction. J Am Coll Cardiol. 1998;32:35–41. 9669246 10.1016/s0735-1097(98)00203-4

[23] Yeboah J , McClelland RL , Polonsky TS , Burke GL , Sibley CT , O'Leary D , Carr JJ , Goff DC , Greenland P , Herrington DM . Comparison of novel risk markers for improvement in cardiovascular risk assessment in intermediate‐risk individuals. JAMA. 2012;308:788–795. 22910756 10.1001/jama.2012.9624 PMC4141475

[24] Biedroń R , Ciszek M , Tokarczyk M , Bobek M , Kurnyta M , Słominska EM , Smoleński RT , Marcinkiewicz J . 1‐Methylnicotinamide and nicotinamide: two related anti‐inflammatory agents that differentially affect the functions of activated macrophages. Arch Immunol Ther Exp (Warsz). 2008;56:127–134. 18373238 10.1007/s00005-008-0009-2 PMC2766500

