# Supplementary material for: Serum N 1‐Methylnicotinamide is Associated With Coronary Artery Disease in Chinese Patients
Source: J Am Heart Assoc. 2017 Feb 8;6(2):e004328. doi: 10.1161/JAHA.116.004328 (PMC5523749; doi:10.1161/JAHA.116.004328)
Supplement: Supplementary file 1 — Data S1. Supplemental methods. Table S1. HPLC Conditions for Measuring N 1‐Methylnicotinamide Table S2. MS Conditions for Measuring N 1‐Methylnicotinamide Table S3. Stability of N 1‐Methylnicotinamide (me‐NAM) Under Different Storage Conditions (n=3) Table S4. Associations of Serum N 1‐Methylnicotinamide Concentration Tertiles With Coronary Artery Disease by Sex Figure S1. Association between serum N 1‐methylnicotinamide and severity of coronary artery disease in men (left) and women (right), respectively. [file JAH3-6-e004328-s001.pdf]

SUPPLEMENTAL MATERIAL

Data S1.

Supplemental Methods

Quantification of serum N^1-methylnicotinamide

Serum me-NAM was measured by liquid chromatography with tandem mass spectrometry (LC/MS/MS) using an electrospray ionization-triple quadrupol mass spectrometer (Agilent G6430, USA) coupled to a liquid chromatography system (Agilent 1290, USA) controlled by MassHunter workstation software with version B. 05.00. Chromatographic separation was achieved on a Spherisorb CNRP 5 µm, 4.6x150 mm analytical column (Waters, USA) at 30 °C with a flow rate of 200 µL/min. A sample volume of 3 µL was injected onto the column. Eluents consisted of 5 mM ammonium formate/0.1% formic acid aqueous solution (A) and 100% acetonitrile (B). The gradient program of the mobile phase was set as follows: a 55/45 (v/v) mixture of solvents A and B from 0.00 to 3.2 min, a 90/10 mixture from 3.3 to 3.6 min, and a 55/45 mixture from 3.7 to 5.5 min, resulting in a total run time of 5.5 min per sample (Table S1). The column flow was directly converted into the electrospray ionization (ESI) source of the mass spectrometer, which was operated in the positive ion mode. The optimal MS parameters were as follows: capillary voltage 4000 V; gas temperature 350 °C, gas flow 10 L/min and nebulizer 20 pounds per square inch (psi). The compound dependent parameters like fragmentor and collision energy for N^1-methylnicotinamide (me-NAM) were optimized at 100 V and 20 V, respectively, and for internal standard N'-methylnicotinamide were 100 V and 25 V, respectively (Table S2). Quantification was performed via peak area ratios (multiple reaction monitoring m/z 137.1 -> m/z 94.1) applied to internal standard N'-methylnicotinamide (multiple reaction monitoring m/z 137.1 -> m/z 80.1) in an external calibration curve.

Samples were prepared using deproteinization with acetonitrile. 20 µL of N'-methy lnicotinamide (internal standard, IS) working solution (30 ng/mL in methanol) and 160 µL of acetonitrile were sequentially added to 50 µL serum sample and vortex-mixed for 2 min. After centrifugation at 12,000 rpm for 10 min at 4 °C, an aliquot of 3 µL of the supernatant was injected into the LC-MS/MS system.

Calibration curves were prepared by spiking pooled blank serum with an appropriate amount of working solution to produce the calibration curve points equivalent to 80, 40, 20, 10, 5, 2.5 ng/mL of me-NAM (Sigma-Aldrich, USA). Samples were made in five replicates and each of them also contained 20 µL of the IS working solution. The results (peak-area ratio of analytes to IS) versus concentration were fitted to the linear equation. The peak intensities of the mean blank serum of me-NAM should be subtracted from the calibration standards response. The results show that the calibration curves were linear over the concentration range of 2.5–80 ng/mL for me-NAM with the linear regression equation of $f = 0.0630 \times C + 0.0268$, $r = 0.9997$ ($n = 5$), where f represented the peak-area ratio of analyte to IS and C represented the serum concentrations of analyte.

The stability of the analytes was validated in analyte-spiked plasma samples under five different storage conditions (Table S3). Three different concentrations of me-NAM (3.75, 15 and 60ng/mL) were used for calculating the variability of the analyte according to the criteria limits defined in FDA Bioanalytical Method Validation Guidance for Industry. The samples for calculating the intra- and inter-assay variability of each concentration of me-NAM is 5 and 15, respectively, resulting in a total sample size of 15 and 45 for calculating the intra- and inter-assay variability, respectively.

Table S1. HPLC Conditions for measuring *N*^{1}-methylnicotinamide

	HPLC Conditions
**Column**	Waters Spherisorb CNRP（4.6×150 mm, 5 μm）
**Column temperature**	30°C
**Mobile phase**	a 55/45 (v/v) mixture of solvents A and B from 0.00 to 3.2 min, a 90/10 mixture from 3.3 to 3.6 min, and a 55/45 mixture from 3.7 to 5.5 min
**Flow rate**	200 μL/min
**Run time**	5.5 min
**Injection volume**	3μL

Table S2. MS Conditions for measuring *N*[1]-methylnicotinamide

	MS Conditions				
**Ion Source**	ESI				
**Polarity**	Positive				
**Scan Type**	MRM				
**Compound name**	Precursor Ion (amu)	Product Ion (amu)	Dwell time (msec)	Fragmentor (v)	Collision energy (v)
N^1-methylnicotinamide	137.1	94.1	200	100	20
N'-methylnicotinamide (IS)	137.1	80.1	200	100	25
**Capillary voltage**	4000 v				
**Gas temperature**	350°C				
**Gas flow**	10 L/min				
**Nebulizer**	20 psi				

ESI indicates electrospray ionization; MRM, multiple reaction monitoring; psi, pounds per square inch.

Table S3. Stability of N^1-methylnicotinamide (me-NAM) under different storage conditions (n=3)

	Spiked concentrations of me-NAM (ng/mL)		
	**3.75**	**15**	**60**
**Condition 1 (as control)**			
Mean ± SD (ng/mL)	3.31 ± 0.14	13.74 ± 0.25	56.40 ± 1.47
RSD (%)	4.28	1.84	2.60
Accuracy (%)	88.37	91.63	93.99
**Condition 2 (4 °C for 24 h)**			
Mean ± SD (ng/mL)	3.40 ± 0.32	14.86 ± 0.34	66.37 ± 1.59
RSD (%)	9.45	2.32	2.40
Accuracy (%)	90.76	99.04	110.62
**Condition 3 (25 °C for 4 h)**			
Mean ± SD (ng/mL)	3.45 ± 0.06	13.78 ± 0.22	58.05 ± 1.61
RSD (%)	1.84	1.57	2.77
Accuracy (%)	92.67	91.47	98.68
**Condition 4 (-20 °C for 25 days)**			
Mean ± SD (ng/mL)	3.53 ± 0.24	13.78 ± 0.14	60.62 ± 2.03
RSD (%)	6.87	1.00	3.34
Accuracy (%)	94.02	91.84	101.04
**Condition 5 (3 freeze/thaw cycles at -20 °C)**			
Mean ± SD (ng/mL)	3.83 ± 0.42	15.22 ± 0.34	62.10 ± 1.13
RSD (%)	10.89	2.25	1.82
Accuracy (%)	102.03	101.46	103.49

Stability of the analytes were validated in analyte-spiked samples under five different storage conditions. Condition 1 represents sample immediately extracted and assayed after the analytes spiked (as control); Condition 2, sample assayed after storage in autosampler at 4 °C for 24 h; Condition 3, sample extracted and assayed after the analytes spiked at 25 °C for 4 h; Condition 4, parallel-prepared sample stored at –20 °C for 25 days; Condition 5, parallel-prepared sample stored after three freeze/thaw cycles at –20 °C. RSD indicates relative standard deviation.

Table S4. Associations of serum *N*[1]-methylnicotinamide concentration tertiles with coronary artery disease by sex

	Serum *N*[1]-methylnicotinamide, ng/ml (Tertile 2 *vs*. Tertile 1)		Serum *N*[1]-methylnicotinamide, ng/ml (Tertile 3 *vs*. Tertile 1)	
	**Odds ratio (95% CI)**	***P***	**Odds ratio (95% CI)**	***P***
**Men (n=193)**				
Crude model	2.19 (1.01–4.75)	0.04	3.07 (1.35–6.96)	0.007
Adjusted model	2.34 (1.01–4.82)	0.04	2.67 (1.06–6.77)	0.03
**Women (n=140)**				
Crude model	2.19 (0.96–5.06)	0.06	3.22 (1.35–7.71)	0.009
Adjusted model	3.31 (1.09–10.09)	0.04	4.99 (1.49–16.72)	0.009

In the adjusted model, odds ratio (95% CI) were adjusted for age, body mass index, systolic blood pressure, current smoking and alcohol intake, hypertension, diabetes, dyslipidemia, use of antihypertensive, antihyperglycemic and hypolipidemic drugs, and fasting plasma glucose, total and LDL cholesterol, and triglycerides.

Figure S1. Association between serum *N*[1]-methylnicotinamide and severity of coronary artery disease in men (left) and women (right), respectively.

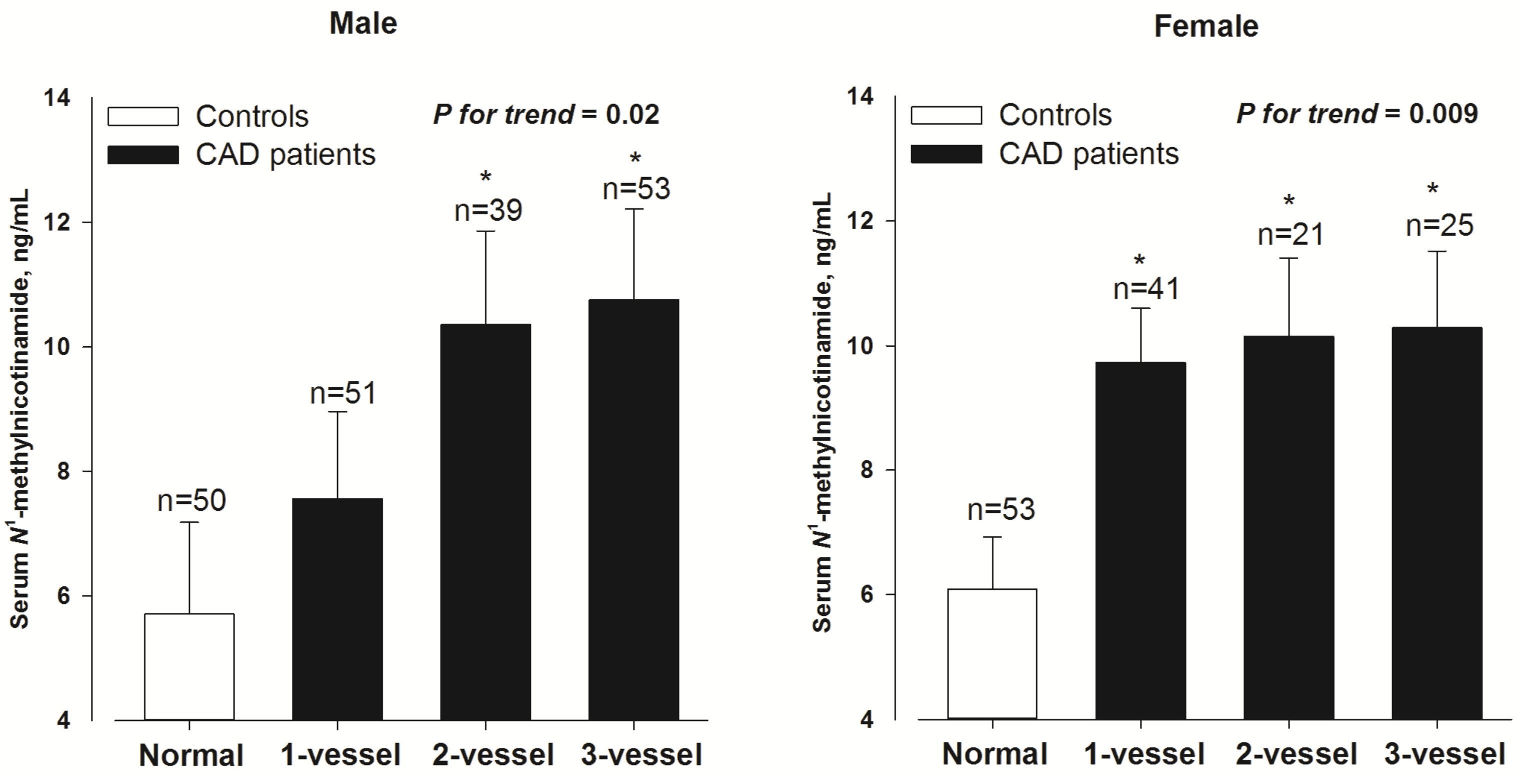

Figure S1. The analysis was adjusted for age, body mass index, systolic blood pressure, current smoking and alcohol intake, hypertension, diabetes, dyslipidemia, use of antihypertensive, antihyperglycemic and hypolipidemic drugs, and fasting plasma glucose, total and HDL

cholesterol, and triglycerides. The *P* value for test for trend of the changes of serum me-NAM concentrations across the severity of coronary angiography is given. * $P \leq 0.01$ vs. normal.
